# Development and Validation of a Risk‐Prediction Nomogram for Preoperative Blood Type and Antibody Testing in Spinal Fusion Surgery

**DOI:** 10.1111/os.13946

**Published:** 2023-12-03

**Authors:** Linghong Wu, Xiaozhong Peng, Xianglong Zhuo, Guangwei Zhu, Xiangtao Xie

**Affiliations:** ^1^ Guangxi Key Laboratory of Orthopaedic Biomaterials Development and Clinical Translation Liuzhou Worker's Hospital Liuzhou China; ^2^ Spine Surgery Liuzhou Worker's Hospital Liuzhou China; ^3^ West Hospital (Orthopaedic Hospital) Liuzhou Worker's Hospital Liuzhou China

**Keywords:** ABO/Rh Type, Antibody Screening, Nomogram, Spinal Fusion Surgery, Transfusion

## Abstract

**Objective:**

With advancements in minimally invasive techniques, the use of spinal fusion surgery is rapidly increasing and transfusion rates are decreasing. Routine preoperative ABO/Rh blood type and antibody screening (T&S) laboratory tests may not be appropriate for all spinal fusion patients. Herein, we constructed a nomogram to assess patient transfusion risk based on various risk factors in patients undergoing spinal fusion surgery, so that preoperative T&S testing can be selectively scheduled in appropriate patients to reduce healthcare and patient costs.

**Methods:**

Patients who underwent spinal fusion surgery between 01/2020 and 03/2023 were retrospectively examined and classified into the training (n = 3533, 70%) and validation (n = 1515, 30%) datasets. LASSO and multivariable logistic regression were used to analyze risk factors for blood transfusion. Nomogram predictive model was built according to the independent predictors and mode predictive power was validated using consistency index (C‐index), Hosmer–Lemeshow (HL) test, calibration curve analysis and area under the curve (AUC) for receiver operating characteristic (ROC) curve. Bootstrap resampling was used for internal validation. Decision curve analysis (DCA) was applied to evaluate the model's performance in the clinic.

**Results:**

Being female, age, BMI, admission route, critical patient, operative time, heart failure, end‐stage renal disease or chronic kidney disease (ESRD or CKD), anemia, and coagulation defect were predictors of blood transfusion for spinal fusion. A prediction nomogram was developed according to a multivariate model with good discriminatory power (C‐index = 0.887); Bootstrap resampling internal validation C‐index was 0.883. Calibration curves showed strong matching between the predicted and actual probabilities of the training and validation sets. HL tests for the training and validation sets had p‐values of 0.327 and 0.179, respectively, indicating good calibration. When applied to the training set, the following parameters were found: AUC: 0.895, 95% CI: 0.871–0.919, sensitivity 78.2%, specificity 86.7%, positive predictive value 29.4% and negative predictive value 98.2%. If the model were applied in the training set, 2911 T&S tests (82.4%) would be eliminated, equaling a RMB349,320 cost reduction. The AUC in the internal validation was: 0.879, 95% CI: 0.839–0.927, sensitivity 75.2%, specificity 88.8%, positive predictive value 34.3%, negative predictive value 97.9%, would eliminate 1276 T&S tests (84.2%), saving RMB 153,120. The DCA curve indicated good clinical application value.

**Conclusion:**

The nomogram based on 10 independent factors can help healthcare professionals predict the risk of transfusion for patients undergoing spinal fusion surgery to target preoperative T&S testing to appropriate patients and reduce healthcare costs.

## Graphical abstract

Our study is the first to combine preoperative baseline patient characteristics, diagnostic and surgical parameters into an innovative, easy‐to‐use risk prediction nomogram for preoperative assessment of transfusion risk in patients undergoing spinal fusion, so that preoperative T&S testing can be selectively scheduled in appropriate patients to reduce healthcare and patient costs.

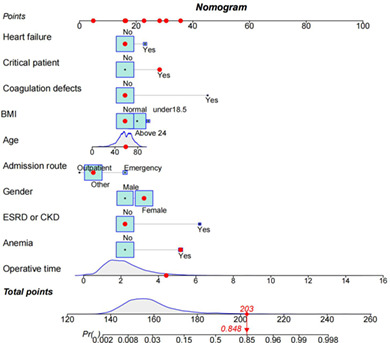

## Introduction

Spinal fusion is an important surgical technique in the treatment of spinal trauma and degenerative disease, whose primary purpose is to stabilize the spine and simultaneously decompress the spinal cord and nerves to relieve pain, promote functional recovery, restore the physiological curvature of the spine, correct deformity, and improve quality of life. Currently, it has become a standard surgery for various spinal conditions, with degenerative conditions being the most common.^1^ The use of spinal fusion surgery is increasing rapidly in the United States. Between 1998 and 2008, the annual number of patients undergoing spinal fusion surgery has risen 2.4 fold (137%), from 174,223 to 413,171 per annum (*p* < 0.001), showing a significantly high rate of increase than other surgeries.^2^ In our hospital, the number of spinal fusion patients increased by ~2.2 fold between 2017 and 2022. Spinal fusion is high cost, with a hospital charge of more than $34,000 on average, not including professional fees.^1^ In today's managed care environment with decreasing reimbursement and increasing costs, proper cost control is critical. Routine preoperative laboratory testing may lead to more tests, consultations, and treatments that ultimately increase costs without reducing adverse events or improving outcomes.^3^, ^4^, ^5^ Abnormal or false‐positive results do not affect anesthesia or perioperative management; however, they can increase costs and patient anxiety.^6^, ^7^ In the age of value‐first medicine, preoperative screening has been justified by citing concerns about patient safety and medico‐legal risks, the perception that other doctors expect preoperative testing, and that the medical benefits outweigh the medical risks, thereby making them cost‐effective. However, evidence suggests that routine laboratory tests are not necessary for screening: studies have shown that in almost 50% of cases, routine laboratory screening (such as routine blood and urine tests, blood grouping, chest X‐ray, and electrocardiogram) is not indicated for some patients.^8^, ^9^, ^10^ In another study, although 12% of clinical tests were abnormal, only 0.5% of abnormal results affected clinical decision‐making.^11^ In another study evaluating preoperative laboratories in patients undergoing posterior spinal fusion for idiopathic scoliosis, Clark et al.^12^ found that 94.9% of patients had one or more abnormal preoperative laboratory values, but only 11.7% of patients took further measure. Nevertheless, preoperative laboratory testing is very expensive, the total annual cost for all specialties in the United States is estimated at $18 billion; unnecessary testing accounted for a large proportion of this, and is considered a major cause of medical waste.^12^

Overall, in recent years the value of traditional preoperative testing has been questioned. Furthermore, laboratory specimens generate a large amount of medical waste that must be disposed of, increasing the environmental burden. As a response to this, the “Best Choice” campaign was jointly implemented by a team of general practitioners and specialists.^13^ Under these precepts, routine preoperative laboratory testing is not recommended for low‐risk procedures such as spinal surgery. At our hospital, preoperative ABO/Rh blood group and antibody screening (T&S) laboratory tests are routinely performed prior to spinal surgery in the event of intraoperative or postoperative blood transfusion. However, the use of computer‐assisted and minimally invasive procedures, new spinal fixation devices, bone graft replacement and complement products (such as bone morphogenetic protein spinal implants and devices), and the use of anti‐fibrinolytic drugs like Tranexamic acid have effectively reduced the amount of intraoperative bleeding and the risk of transfusion in spinal surgery patients.^1^, ^14^ Between 2011 and 2015, the rate of perioperative blood transfusion in spinal surgery decreased from 16.0% to 8.7% over the past decade.^15^ In the last 6 years, our hospital's spinal fusion blood transfusion rate is about 6.0%, which is low.

Given these trends, the controversial value of preoperative testing, and our commitment to reducing unnecessary testing, we hypothesize that not all spinal fusion patients require routine preoperative T&S testing, and that it would therefore be necessary to screen for those who do not require T&S testing. In addition, this type of research is currently rare. Therefore, this study reviewed the data of patients undergoing spinal fusion surgery at our hospital and analyzed the impact of these factors on the risk of transfusion in patients with spine fusion surgery. Therefore, the purpose of this study was: (i) to analyze the independent risk factors for blood transfusion in patients undergoing spinal fusion；and (ii) to develop a predictive model to assess patient risk and the medical validity of preoperative T&S testing in order to selectively schedule preoperative T&S testing in appropriate patients, and to reduce medical costs and patients' financial burden.

## Methods

### Patients and Study Design

The study program was approved by Liuzhou Worker's Hospital Ethics Committee (Liuzhou, Guangxi, China) (ethics number: KY2022073), and was conducted in agreement with Declaration of Helsinki (2013 version). All patients provided written consent to participate.

**FIGURE 1:**
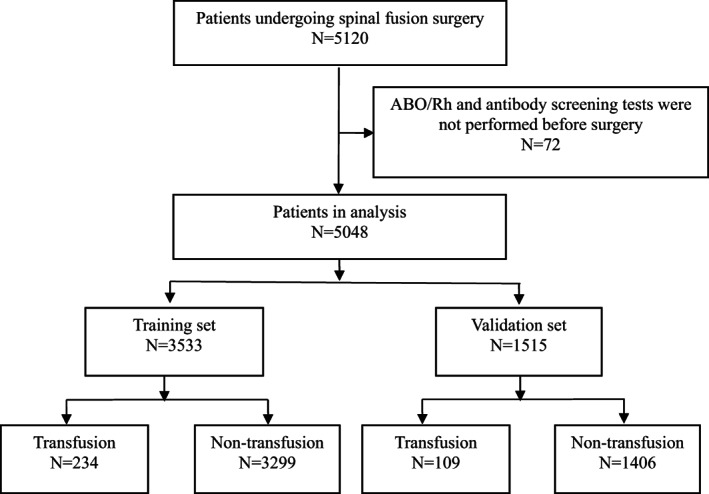
Of the 5048 patients who underwent spinal fusion, 72 were excluded (n = 72), 3533 (70%) were enrolled in the training set, and 1515 (30%) were enrolled in the validation set.

In this retrospective study, we analyzed patients undergoing spinal fusion surgery at our hospital between the 1st January, 2020, and the 31st March, 2023. Inclusion criteria: (i) all surgical procedures were performed by spinal fusion; (ii) complete case information and perioperative records were available; (iii) no contraindications to surgical treatment; Exclusion criteria: (i) no preoperative ABO/Rh and antibody screening tests; (ii) history of spinal radiotherapy; (iii) incomplete clinical data. All of the data were extracted from our electronic medical record system. Patients who underwent spinal fusion surgery (ICD‐9‐CM‐3 = 81.0) during the study period were initially searched using the International Classification of Surgery ICD‐9‐CM‐3 code, with data extraction and entry performed by two investigators, with a third investigator making the final decision on data in case of disagreement. All data collectors were trained in adjudication and data collection. A total of 5120 patients were enrolled. After excluding 72 subjects who had not undergone preoperative ABO/Rh and antibody screening, 5048 subjects were finally included in the study; of these, 70% were randomly assigned to the training set and 30% to the validation set (Figure 1).

### Data Collection

The data source was the inpatient records of patients in the electronic medical record system version 3.5 (ZOE SOFT, China), and the required indicators were obtained using a combination of database technology to mine information from medical records and manual reading of medical records. All the data obtained were manually reviewed by two doctors from the spinal surgery department of Liuzhou Worker's Hospital.

Patients were classified according to age, gender, marital status, nationality, body mass index (BMI), admission route, number of hospitalizations, surgical history, history of drug allergies, critical patients, educational level, case stage, occupation, blood type, comorbidities (diabetes, hypertension, anemia, coagulation disorder, etc.), surgical procedures, anesthetic type, American Society of Patients Anesthesiologists [ASA] score, operative time, and number of bones fused, operator title, all of which were classified as predictive variables, while intraoperative or postoperative blood transfusion were used as the outcome indices.

### Correlation Index Definition

Blood transfusion, or allogeneic transfusion, is defined as the safe transfusion of blood or blood components from another person (usually a donor) with the same blood type as the patient, as needed. Our hospital follows the guidelines of the American Society of Anesthesiologists (ASA), which states that if the patient's hemoglobin concentration (Hb) is more than 10 g/dL, no red blood cell transfusion is required. If Hb is less than 6 or 7 g/dL, transfusion should be considered. If Hb is between 6 and 10 g/dL, red cell transfusion should be considered based on the patient's body metabolism, cardiopulmonary compensatory capacity, and oxygen consumption.^16^ For postoperative transfusion, our transfusion threshold follows the Association for the Advancement of AABB guidelines of 8 g/dL.^17^, ^18^ Comorbidities are classified by the International Classification of Diseases, 10th Revision (ICD‐10) diagnosis codes that are used in the preoperative diagnosis in the surgical record, and surgical procedures are defined by the ICD‐9‐CM‐3 surgical procedure codes, which are included in the surgical name in the surgical record (Table 1). According to China's title evaluation system, the level of operator title is divided into chief, deputy chief, intermediate and primary, of which the title of deputy chief requires at least 10 years of professional and technical work; the title of chief physician requires 5 years of work as a deputy chief physician, that is, at least 15 years of professional and technical work.

**TABLE 1:** Comorbidities definition according to ICD‐10, surgery definition according to ICD‐9‐CM‐3.

Comorbidity	ICD‐10code	Surgery	ICD‐9‐CM‐3 code
Diabetes	E10–E14	Spinal fusion not otherwise specified	81.00
Sleep apnea syndrome	G47	Atlanto‐axial spinal fusion	81.01
COPD	J44	Other cervical fusion of the anterior column, anterior method	81.02
Cardiac arrhythmias	I44–I49	Other cervical fusion in the posterior column, posterior method	81.03
Heart failure	I11, I13.0, I13.2, I50	Dorsal and dorsal lumbar fusion of the anterior column, anterior method	81.04
CAD	I20–I25	Dorsal and dorsolumbar fusion, posterior method	81.05
ESRD or CKD	N18, I12, I13	Lumbar and lumbosacral fusion in the anterior column, anterior meth	81.06
Anxiety or Depression	F32, F33, F41	Lumbar and lumbosacral fusion of the posterior column, posterior method	81.07
Hypertension	I10–I15	Lumbar and lumbosacral fusion in the anterior column, posterior method	81.08
Peripheral vascular disease	I70–79, I80–I89	4–8 segmental fusion	81.63
Anemia	D50–D53, D55–D59, D60–D64	2–3 segmental fusion	81.62
Coagulation defects or purpura or hemorrhagic disorders	D65–D69		
Viral hepatitis	B15–B19		
Diseases of the liver	K70–K77		
Malignant neoplasms	C00–C97		

### Statistical Analysis

All statistical analyses and graphs were performed/drawn using R software, ver. 4.2.3 (R Foundation for Statistical Computing, Vienna, Austria). Continuous data conforming to the standard distribution were presented as the mean (±standard deviation) and the difference between both groups was analyzed using t‐tests. Data not conforming to normal distribution were presented using the median (percentile), and the difference between the two groups was analyzed using the rank‐sum test. Classification data were expressed as numbers and percentages or constituent ratios. Chi‐squared test (or Fisher's exact probability method) was applied to analyze the difference in classification variable distributions between the two groups. Predictors were screened by lasso regression according to the variables identified on univariate analysis, and independent risk factors were identified by logistic regression. Predictive model of transfusion risk in patients undergoing spinal fusion based on independent risk factors and presented in a nomogram, and the probability of transfusion risk was calculated based on the nomogram. To evaluate the model of power, the C‐index was calculated and the Hosmer‐Lemeshow (HL) goodness‐of‐fit statistical test was applied to draw the calibration curve of the model to further predict its accuracy, while a receiver operating characteristic (ROC) curve was drawn to further reflect the sensitivity and specificity of the model. Internal validation was performed using bootstrap resampling with 1000 replicates. To determine the clinical value of the model, a decision curve analysis (DCA) was constructed to measure the net benefit of the model below a given threshold. Statistical significance was defined as a two‐tailed *p* value < 0.05.

## Results

### Number of Spinal Fusion Surgery Cases and Transfusion Rate Changes

**FIGURE 2:**
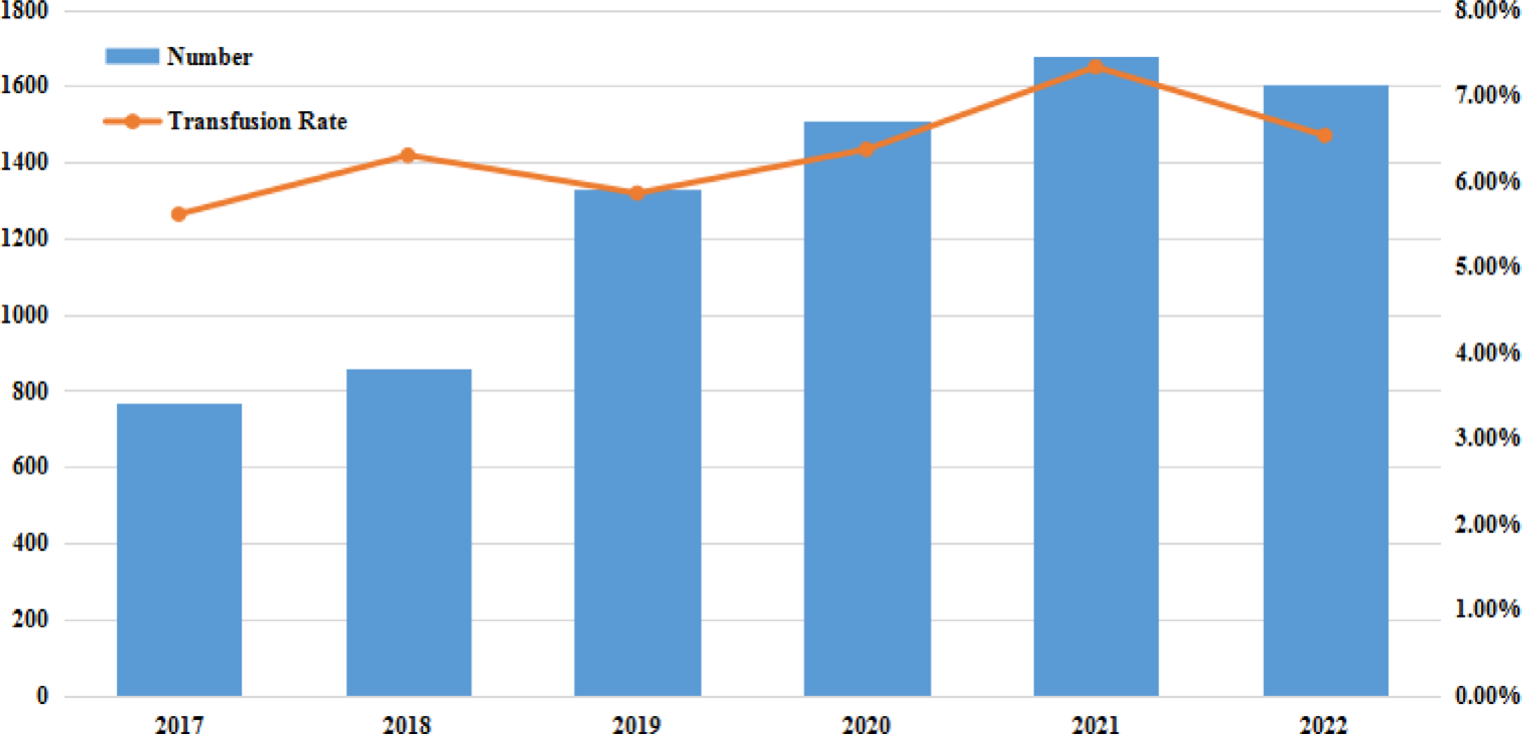
Basic information of spinal fusion surgery and blood transfusion from 2017 to 2022.

In this study, we retrospectively analyzed patients who underwent spinal fusion surgery at our hospital in the last 6 years, finding a mortality rate of 0.00%. As such, we considered spinal fusion surgery to be an effective and safe low‐risk treatment. The number of spinal fusion surgeries from 2017 to 2022 showed an increasing trend, from 756 in 2017 to 1607 in 2022, an increase of 121.83%. However, the transfusion rate remained at around 6% (Figure 2).

### Clinical Characteristics of the Study Subjects

The study involved a total of 5048 cases, of whom 343 (6.79%) received a blood transfusion and 4705 (93.21%) did not. Clinical characteristics included age, gender, number of hospitalizations, history of drug allergy, history of surgery, critical patients, case stage, education, BMI, marital status, occupation, admission route, diabetes, heart failure, CAD, ESRD or CKD, hypertension, peripheral vascular disease, anemia, coagulation defect, use of TXA, type of surgery, ASA ≥3, operative time, surgical procedures, 4–8 segmental fusion and number of preoperative diagnoses statistical difference between transfusion and no transfusion group. No statistical differences were found for any of the other indicators (*P* > 0.05), as shown in Table 2.

**TABLE 2:** Clinical characteristics of the subjects.

Value	All cases (n = 5048)	No transfusion (n = 4705)	Transfusion (n = 343)	*p*‐value
Age (mean (SD), years)	57.72 (12.43)	57.47 (12.10)	61.23 (15.85)	<0.001
Number of hospitalizations (median [IQR])	1.00 [1.00, 1.00]	1.00 [1.00, 1.00]	1.00 [1.00, 2.00]	<0.001
Gender/female (%)	2712 (53.7)	2490 (52.9)	222 (64.7)	<0.001
History of drug allergy (%)	273 (5.4)	241 (5.1)	32 (9.3)	0.001
History of surgery (%)	1407 (27.9)	1288 (27.4)	119 (34.7)	0.004
Critical patients (%)	138 (2.7)	86 (1.8)	52 (15.2)	<0.001
Case stage (C + D) (%)	946 (18.7)	862 (18.3)	84 (24.5)	0.006
Education (%)				<0.001
Primary school and below	2722 (53.9)	2563 (54.5)	159 (46.4)	
Junior to senior	1191 (23.6)	1070 (22.7)	121 (35.3)	
College and above	1027 (20.3)	966 (20.5)	61 (17.8)	
Other	108 (2.1)	106 (2.3)	2 (0.6)	
BMI (kg/m^2^)				<0.001
18.5–23.9	2437 (48.3)	2328 (49.5)	109 (31.8)	
Under 18.5	92 (1.8)	80 (1.7)	12 (3.5)	
Above 24	2519 (49.9)	2297 (48.8)	222 (64.7)	
Nationality (%)				0.454
Han ethnic group	3185 (63.1)	2977 (63.3)	208 (60.6)	
Zhuang ethnic group	1536 (30.4)	1417 (30.1)	119 (34.7)	
Dong ethnic group	103 (2.0)	98 (2.1)	5 (1.5)	
Miao ethnic group	74 (1.5)	71 (1.5)	3 (0.9)	
Yao ethnic group	64 (1.3)	60 (1.3)	4 (1.2)	
Other	86 (1.7)	82 (1.7)	4 (1.2)	
Marital status (%)				0.048
Unmarried	68 (1.3)	58 (1.2)	10 (2.9)	
Married	3792 (75.1)	3547 (75.4)	245 (71.4)	
Bereaved spouse	3 (0.1)	3 (0.1)	0 (0.0)	
Divorce	5 (0.1)	4 (0.1)	1 (0.3)	
Other	1180 (23.4)	1093 (23.2)	87 (25.4)	
Occupation (%)				<0.001
Workers	2021 (40.0)	1903 (40.4)	118 (34.4)	
Retirees	860 (17.0)	781 (16.6)	79 (23.0)	
Business services	837 (16.6)	803 (17.1)	34 (9.9)	
Household and non‐working	448 (8.9)	408 (8.7)	40 (11.7)	
Farmers	657 (13.0)	604 (12.8)	53 (15.5)	
Other	225 (4.5)	206 (4.4)	19 (5.5)	
Admission route (%)				<0.001
Outpatient	4756 (94.2)	4472 (95.0)	284 (82.8)	
Emergency	273 (5.4)	216 (4.6)	57 (16.6)	
Other	19 (0.4)	17 (0.4)	2 (0.6)	
Blood type (%)				0.115
A	1227 (24.3)	1127 (24.0)	100 (29.2)	
B	1367 (27.1)	1279 (27.2)	88 (25.7)	
AB	302 (6.0)	285 (6.1)	17 (5.0)	
O	2115 (41.9)	1977 (42.0)	138 (40.2)	
Other	37 (0.7)	37 (0.8)	0 (0.0)	
Diabetes (%)	511 (10.1)	465 (9.9)	46 (13.4)	0.046
Sleep apnea syndrome (%)	2 (0.0)	2 (0.0)	0 (0.0)	1.000
COPD (%)	12 (0.2)	10 (0.2)	2 (0.6)	0.432
Cardiac arrhythmias (%)	252 (5.0)	227 (4.8)	25 (7.3)	0.058
Heart failure (%)	194 (3.8)	161 (3.4)	33 (9.6)	<0.001
CAD (%)	143 (2.8)	126 (2.7)	17 (5.0)	0.022
ESRD or CKD (%)	112 (2.2)	43 (0.9)	69 (20.1)	<0.001
Anxiety or depression (%)	25 (0.5)	23 (0.5)	2 (0.6)	1.000
Hypertension (%)	1270 (25.2)	1161 (24.7)	109 (31.8)	0.004
Peripheral vascular disease (%)	566 (11.2)	502 (10.7)	64 (18.7)	<0.001
Anemia (%)	271 (5.4)	163 (3.5)	108 (31.5)	<0.001
Coagulation defects or purpura or hemorrhagic disorders (%)	26 (0.5)	7 (0.1)	19 (5.5)	<0.001
Viral hepatitis (%)	145 (2.9)	135 (2.9)	10 (2.9)	1.000
Diseases of the liver (%)	1086 (21.5)	1026 (21.8)	60 (17.5)	0.070
Malignant neoplasms (%)	115 (2.3)	109 (2.3)	6 (1.7)	0.622
Use of TXA (%)	3411 (67.6)	3199 (68.0)	212 (61.8)	0.021
Operation type/elective surgery (%)	4775 (94.6)	4489 (95.4)	286 (83.4)	<0.001
ASA ≥ 3	816 (16.2)	715 (15.2)	101 (29.4)	<0.001
Operative time (mean (SD))	2.22 (1.10)	2.14 (1.02)	3.33 (1.57)	<0.001
Surgical procedures (%)				<0.001
Spinal fusion not otherwise specified	68 (1.3)	52 (1.1)	16 (4.7)	
Atlanto‐axial spinal fusion	20 (0.4)	15 (0.3)	5 (1.5)	
Other cervical fusion of the anterior column, anterior method	836 (16.6)	807 (17.2)	29 (8.5)	
Other cervical fusion in the posterior column, posterior method	104 (2.1)	88 (1.9)	16 (4.7)	
Dorsal and dorsal lumbar fusion of the anterior column, anterior method	36 (0.7)	26 (0.6)	10 (2.9)	
Dorsal and dorsolumbar fusion, posterior method	149 (3.0)	121 (2.6)	28 (8.2)	
Lumbar and lumbosacral fusion in the anterior column, anterior method	883 (17.5)	817 (17.4)	66 (19.2)	
Lumbar and lumbosacral fusion of the posterior column, posterior method	2783 (55.1)	2614 (55.6)	169 (49.3)	
Lumbar and lumbosacral fusion in the anterior column, posterior method	169 (3.3)	165 (3.5)	4 (1.2)	
4–8 segmental fusion (%)	236 (4.7)	202 (4.3)	34 (9.9)	<0.001
Number of preoperative diagnoses (median [IQR])	3.00 [2.00, 5.00]	3.00 [2.00, 5.00]	6.00 [4.00, 9.50]	<0.001
Operator title				0.068
Chief physician	3644 (72.2)	3411 (67.6)	233 (4.6)	
Deputy chief physician	1404 (27.8)	1294 (27.5)	110 (2.2)	

Abbreviations: COPD, chronic obstructive pulmonary disease; CAD, coronary artery disease; ESRD or CKD, end‐stage renal disease or chronic kidney disease; TXA, tranexamic acid.

### Lasso Regression Screening Variables

**FIGURE 3:**
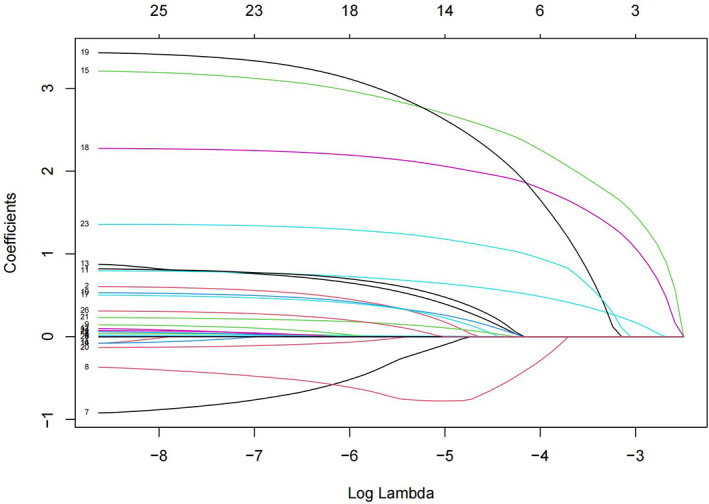
LASSO regression coefficient variation characteristic.

**FIGURE 4:**
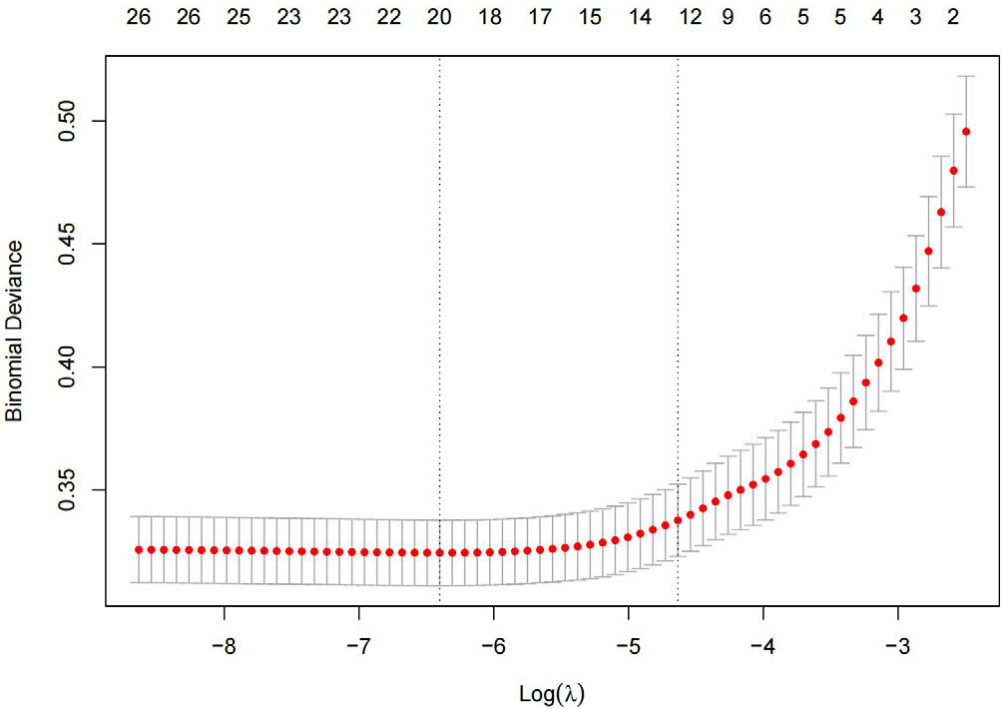
We choose the most appropriate parameter lambda value (λ = 0.009716439) by cross‐validation for LASSO regression, and the dashed line on the left indicates the value of lambda that represents the average value of the minimum target parameter. Under these conditions, the model achieved optimal performance.

To minimize the impact of covariates on the variables weighted for this indicator within the linear model, all were variables identified as statistically different on the univariate analysis were screened using Lasso regression, yielding 12 variables for use as independent variables in subsequent multifactorial analyses at λ = 0.009716439 (Figures 3 and 4). These variables were gender, age, BMI, route of admission, critical patients, ASA classification, operative time, heart failure, ESRD or CKD, anemia, coagulation defect, and the use of TXA, with the remaining indicators found not to be possible risk factors affecting blood transfusion.

### Multivariate Analysis

Multivariate logistic regression revealed that female gender (OR:2.955, 95% CI: 2.034–4.340, *p* < 0.0001), age (OR: 1.019, 95% CI: 1.004–1.034, *p* < 0.0001), outpatient admission (OR:0.304, 95% CI: 0.175–0.538, *p =* 0.0131), BMI ≤18.5 (OR: 2.726, 95%CI: 0.9156–7.114, *p* = 0.030), BMI ≥24 (OR: 1.825, 95% CI: 1.273–2.640, *p* = 0.0004), critical patient (OR: 3.918, 95%CI: 2.043–7.410, *p* < 0.0001), operation time (OR: 2.390, 95%CI: 2.101–2.729, *p* < 0.0001), heart failure (OR: 3.141, 95%CI: 1.632–5.792, *p* = 0.0004), ESRD or CKD (OR: 24.343, 95% CI: 13.182–45.402, *p* < 0.0001), anemia (OR: 9.364, 95% CI: 6.044–14.466, *p* < 0.0001), and coagulation defects (OR: 52.639, 95% CI: 14.361–223.502, *p* < 0.0001) were all independent and significant predictors of blood transfusion for spinal fusion surgery (Table 3).

**TABLE 3:** A multivariate logistic regression analysis of risk factors for blood transfusion in spinal fusion surgery.

Variable	*β*	SE	OR	95%CI	*p* value
Female	1.081	0.193	2.955	2.034–4.340	<0.0001
Age	0.018	0.007	1.019	1.004–1.034	<0.0001
Admission route/outpatient	−1.192	0.287	0.304	0.175–0.538	0.0131
BMI ≤ 18.5	1.003	0.520	2.726	0.9156–7.114	0.030
BMI ≥ 24	0.602	0.186	1.825	1.273–2.640	0.0004
Critical patient	1.366	0.328	3.918	2.043–7.410	<0.0001
Operative time	0.871	0.067	2.390	2.101–2.729	<0.0001
Heart failure	1.144	0.322	3.141	1.632–5.792	0.0004
ESRD or CKD	3.192	0.315	24.343	13.182–45.402	<0.0001
Anemia	2.237	0.222	9.364	6.044–14.466	<0.0001
Coagulation defects	3.963	0.684	52.639	14.361–223.502	<0.0001

### Development and Validation of the Nomogram

**FIGURE 5:**
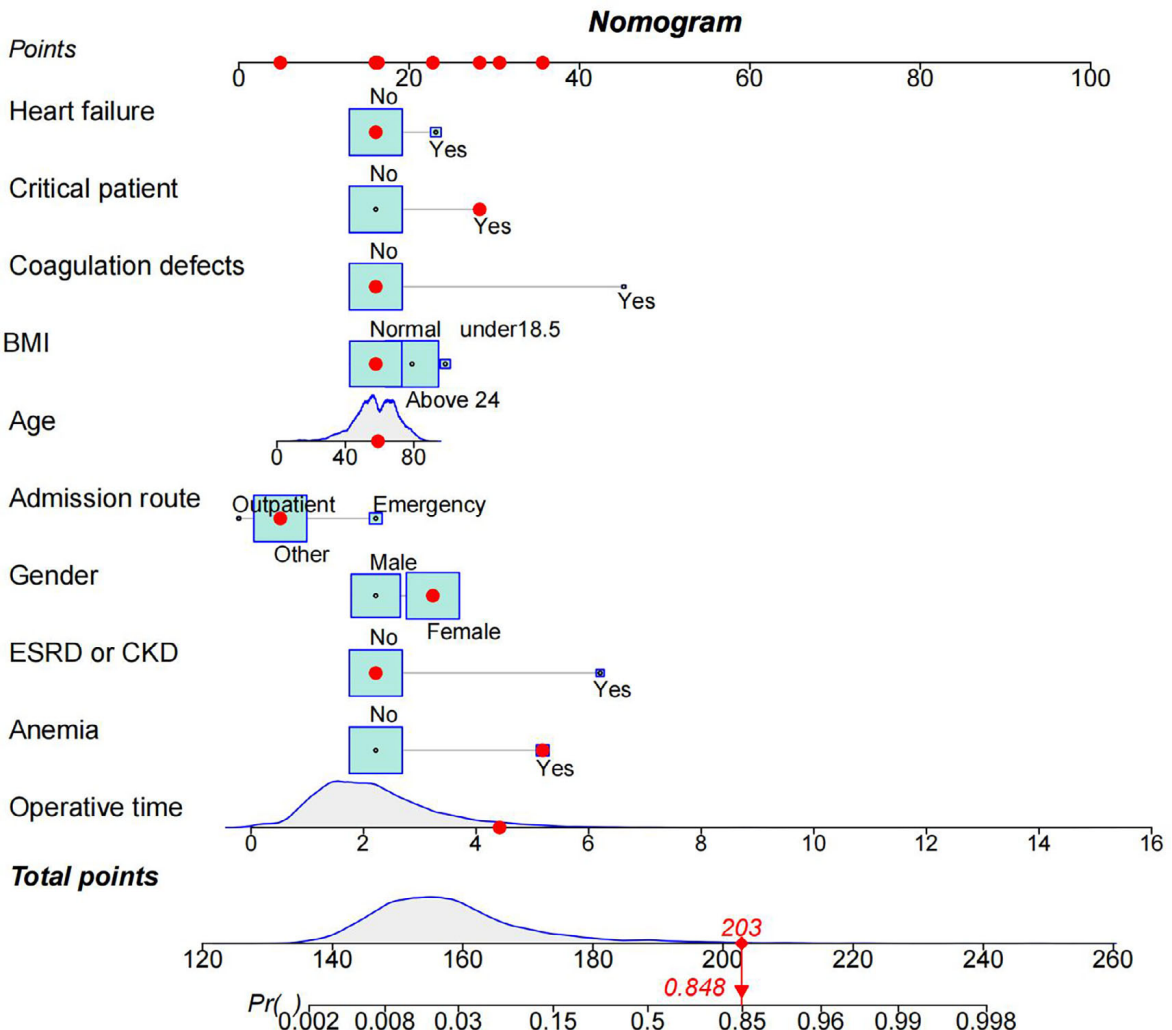
Nomogram for the prediction of blood transfusion for spinal fusion. The red dots in the figure indicate nomogram usage. Projecting the patient's information onto the nomogram, the patient ended up with a score of 203, corresponding to a probability of 0.848 (>0.5), so we judged that the patient would eventually need a blood transfusion. The patient also eventually performed a transfusion in accordance with our prediction.

In order to develop clinical tools to assist clinicians in predicting surgical blood transfusion in patients, preoperative screening was selectively scheduled in appropriate patients. The transfusion risk nomogram prediction model was then plotted using the findings of multivariate logistic regression analysis, with a nomogram C‐index of 0.887, indicating that the model has a high level of discriminatory power (Figure 5). After 1000 bootstrap resampling validation, it was found that the C‐index was 0.883, which was evidence that the discriminative power of the model was excellent.

**FIGURE 6:**
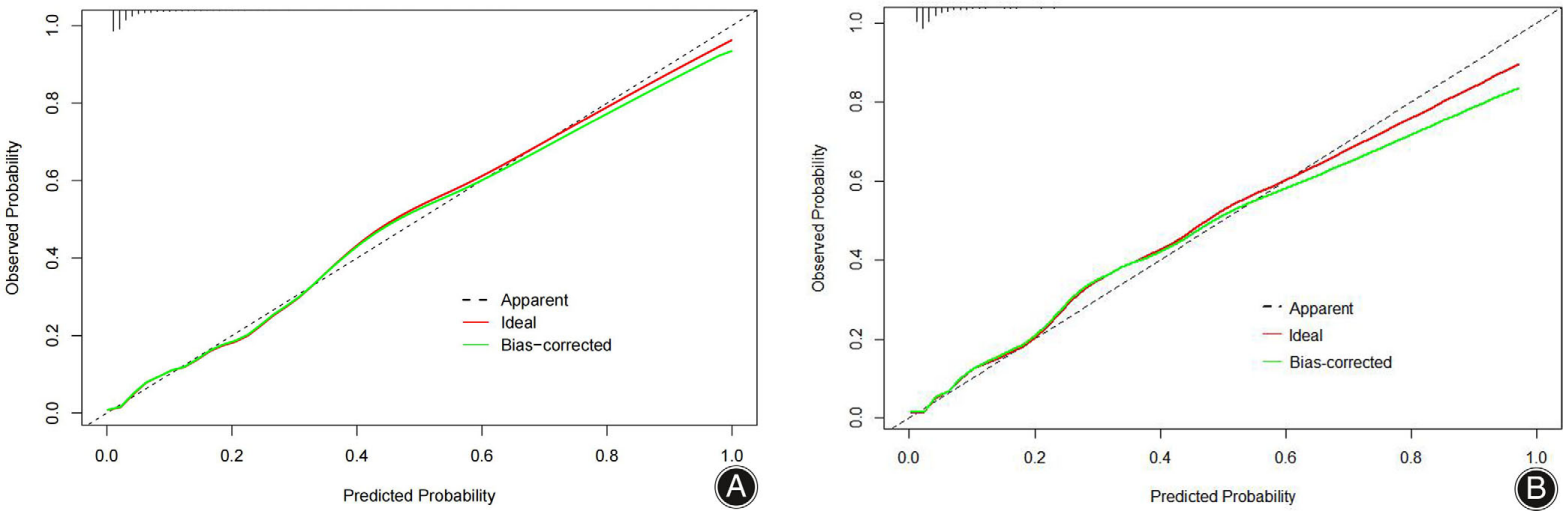
Calibration plots of the nomogram of the training set (A) and validation set (B).

**FIGURE 7:**
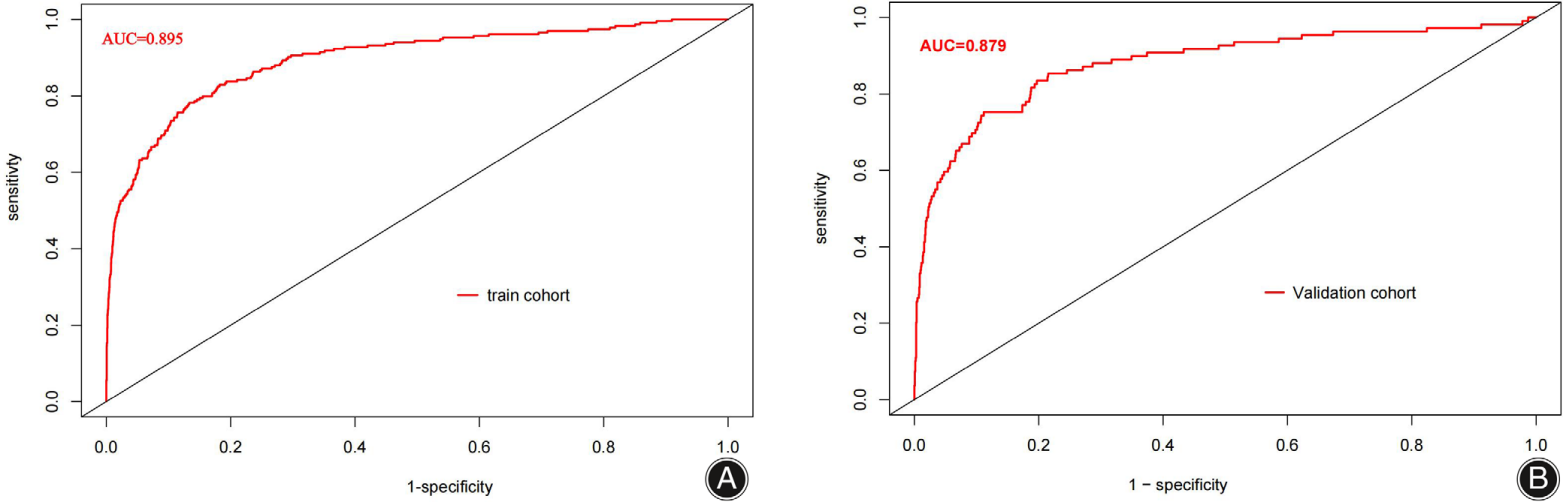
Receiver operating characteristic analysis of the training set (A) and validation set (B).

**FIGURE 8:**
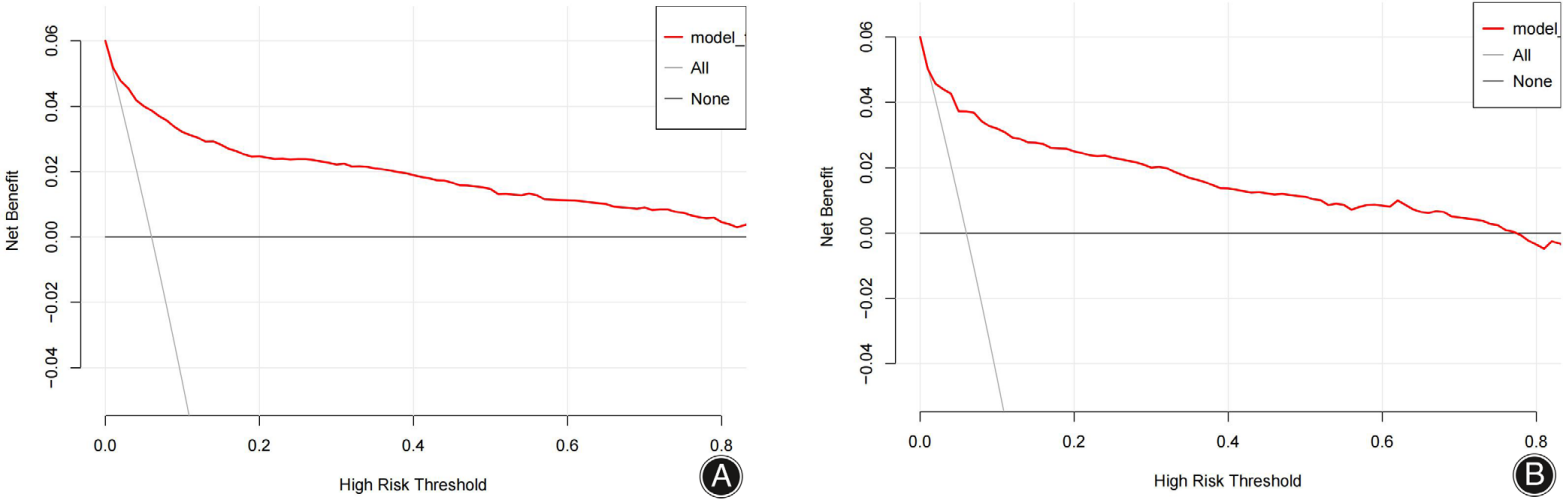
Decision curve analysis of the nomogram model of the training set (A) and validation set (B).

The consistency of the predicted and real results was assessed by plotting calibration curves for both the training and validation sets (Figure 6A/B). In addition, the Hosmer–Lemeshow goodness‐of‐fit tests for the training and validation sets were χ^2^ = 9.1902 (*p* = 0.327) and χ^2^ = 11.421 *(p* = 0.179), respectively, with *p*‐values that were not significant on either the training or validation sets, indicating that the nomogram plots were well‐fitted. The ROC curve results showed an AUC of 0.895 (95% CI: 0.871–0.919; Figure 7A), a sensitivity of 78.2%, a specificity of 86.7%, a positive predictive value of 29.4%, and a negative predictive value of 98.2%. In other words, if the model were applied in clinical practice in the training set, 2911 T&S tests (82.4%) would be eliminated, resulting in a cost saving of RMB 349,320 (Table 4). The internal validation results showed an AUC under the ROC curve of 0.879 (95% CI: 0.839–0.927; Figure 7B), a sensitivity of 75.2%, a specificity of 88.8%, a positive predictive value of 34.3%, and a negative predictive value of 97.9%, which means that if the model were applied in the test set in clinical practice, 27 (1.78%) patients requiring blood transfusion would not receive preoperative T&S, and 1276 T&S tests (84.2%) would have been eliminated, resulting in a cost saving of RMB 153,120 (Table 4). The DCA demonstrated that the prediction of the nomogram plot has a high level of clinical applicability (Figure 8A/B).

**TABLE 4:** Application of risk‐based nomogram to training and verification cases.

Data	n	Transfusions not meeting criteria (N, % Rate)	Transfusions meeting criteria (N, % Rate)	Sensitivity (%)	Specificity (%)	Positive predictive value (%)	Negative predictive value (%)	Potential type & screens eliminated (N, % Rate)	Cost per type & screen (yuan)	Total cost savings (yuan)
Train	3533	51 (1.01%)	183 (5.18%)	78.2	86.7	29.4	98.2	2911 (82.4%)	120	349,320
Validation	1515	27 (1.78%)	82 (5.41%)	75.2	88.8	34.3	97.9	1276 (84.2%)	120	153,120

## Discussion

In the present study, we developed a nomogram using 10 preoperative predictors and validated it for use as a predictor of the risk of blood transfusion in spinal fusion patients. Using this nomogram, we found that approximately 98% of patients who do not require intraoperative or postoperative blood transfusion can be accurately identified in the hospital's data set, further enabling personalized patient consultation and decision‐making in the management of preoperative routine T&S screening tests for spinal surgery patients, so that preoperative T&S screening tests may be selectively scheduled in appropriate patients, and unnecessary preoperative testing can be reduced. Such a reduction of medical costs would further reduce the financial burden on patients.

### Risk Factors for Blood Transfusion Risk

If a risk‐based nomogram selective screening method is to be used for T&S testing, the capability to accurately identify patients at highest risk for transfusion is critical. Herein, we identified 10 independent preoperative factors to help predict transfusion risk. Coagulation defect (OR = 52.639, 95% CI: 14.361–223.502, *p* < 0.0001) is a significant predictor of blood transfusion in spinal fusion patients, and compared to patients with normal coagulation, patients with coagulation defect are weighted by about 28 points in the nomogram. In this condition, coagulation factors fail to stop bleeding in time, resulting in excessive blood loss, and requiring the need for hemostasis. A recent study also showed that coagulation defects are an important predictor of intraoperative or postoperative transfusion in spine surgery.^19^ Further, we found that comorbid end‐stage renal disease or chronic kidney disease (OR = 24.343, 95% CI: 13.182–45.402, *p* < 0.0001), anemia (OR = 9.364, 95% CI: 6.044–14.466, *p* < 0.0001), and heart failure (OR = 3.141, 95% CI: 1.632–5.792, *p* = 0.0004) were also independent risk factors for transfusion in patients, and while patients with end‐stage renal disease or chronic kidney disease, patients with anemia, and patients with heart failure would have an increased weighting of approximately 25, 18, and 6 points, respectively, in the nomogram. The association between chronic renal insufficiency and an increase in blood transfusion has been reported and recently confirmed by several studies, including that by Vora et al.,^20^ who showed an increased risk of bleeding, blood transfusion, and dialysis after cardiac catheterization in patients with chronic kidney disease (CKD). Ibanez et al.^21^ similarly reported an increased transfusion and prolonged hospital stay in patients undergoing cardiac valve surgery with mild preoperative renal insufficiency (measured as GFR ≤ 60 mL/min/1.73 m^2^). The present study adds to and extends this view, suggesting that this association exists not only in cardiac surgery, but also in spinal surgery. The underlying mechanisms may involve impaired platelet function and platelet endothelial interaction,^22^ among a number of other factors, including abnormalities in coagulation and fibrinolysis, inflammation and oxidative stress, reduced nitric oxide synthesis, inadequate erythropoietin production, anemia, uremic toxin accumulation and malnutrition, among others, which have also been suggested as causes of excessive blood transfusion in patients undergoing surgery for chronic kidney disease.^22^, ^23^, ^24^ The potential mechanism between heart failure and blood transfusion may be related to increased inflammatory factors such as TNF‐α, IL‐1, IL‐6, and IL‐13 in heart failure patients, which cause disturbance in the homeostasis of iron metabolism in vivo, impaired proliferation of red lineage progenitor cells, reduced production and weakened effect of EPO, impaired metabolism and utilization of iron caused by iron‐regulating hormone (hepcidin), etc., thus causing anemia, as well as malnutrition anemia caused by heart failure gastrointestinal edema, gastrointestinal stasis causing impaired absorption of iron, folic acid, vitamin B12, and other hematopoietic raw materials. Blood transfusion is commonly used as an early treatment to solve this difficult problem. Patients with multiple illnesses are more prone to taking drugs that influence blood clotting and have a greater risk of anemia, which may explain this phenomenon.^25^ In short, patients and doctors need to be aware of these elevated risks.

In the present study, we found that female gender (OR: 2.955, 95%CI: 2.034–4.340, *p* < 0.0001), age (OR: 1.019, 95%CI: 1.004–1.034, *p* < 0.0001), outpatient admission (OR: 0.304, 95%CI: 0.175–0.538, *p* = 0.0131), BMI ≤ 18.5 (OR: 2.726, 95%CI: 0.9156–7.114, *p* = 0.03), BMI ≥ 24 (OR: 1.825, 95% CI: 1.273–2.640, *p* = 0.0004), critical patients (OR: 3.918, 95% CI: 2.043–7.410, *p* < 0.0001), and operation time (OR: 2.390, 95%CI: 2.101–2.729, *p* < 0.0001) were independent and significant predictors of postoperative or intraoperative blood transfusion in spinal fusion surgery. As can be seen from the nomogram, females, BMI ≤ 18.5, emergency admissions, and critically ill patients increase the weighting by approximately 6, 8, 15, and 12 points, respectively, whereas each 10‐year increase in age increases the score in the predictive model by approximately 2 points, and each hour increase in operative hour rate increases the score in the predictive model by approximately 6 points. A prior study of 54,122 transfusion patients showed that the transfusion rate in female patients was almost three times higher than in male patients.^26^ This finding is consistent with that of Song et al.,^27^ and may be due to clinicians using the same absolute transfusion strategy and the performance of a liberal transfusion strategy in the clinical setting.^28^ In this study, age was a predictor of transfusion in spinal surgery, with a higher transfusion frequency in older patients. Older patients have increased vascular fragility and lower coagulation factor activity than younger patients, leading to increased surgical bleeding. In a prior study, Duncan et al.^29^ used the data of 378 primary total elbow arthroplasties to construct a predictive model for transfusion in spinal surgery and found that age was a significant influencing factor. These results agree with those of Ahmadi et al.^30^ In addition, the current study suggests that a lower BMI is a predictor of transfusion risk in spinal fusion surgery. The ability of a lower BMI to predict the risk of transfusion increased progressively, prolonging the duration of surgery and thereby increasing the transfusion risk. These findings are in agreement with those of Liu et al.^31^ Overall, we postulate that in complex and difficult surgery, effective communication among the surgical team, surgeons, theater nurses, and anesthetists can improve operating time and reduce the rate of transfusion.

It is interesting to note that the application of tranexamic acid was not identified as a factor independently predicting the need for blood transfusion in this study. However, the application of tranexamic acid in the perioperative period of minor orthopaedic surgery has been reported to be effective at reducing blood loss, transfusion rates, and complication rates.^32^ Unfortunately, the administration of tranexamic acid can lead to varying results, and there is currently no consensus on the optimal mode of treatment with tranexamic acid.^33^ Future studies are needed to further investigate the dose, route, and timing of administration.

### Clinical Significance of the Study

This study further developed a transfusion risk line graph prediction model based on the above transfusion risk factors, which can predict the risk of transfusion in patients before surgery. After the model was constructed, it was tested for discrimination and accuracy. The C‐index estimated the probability that the model prediction would be consistent with the actual observed outcome. In this study, the the model and internal validation C‐index was 0.887 ≥ 0.60, indicating that the model can be used as a strong guide to predict surgical transfusion in advance. Accuracy reflects the degree of agreement between predicted and actual risk, with *p* > 0.05 for the Hosmer–Lemeshow goodness‐of‐fit test indicating a good fit between the model prediction and the actual situation.

Furthermore, the training and validation set DCA curves indicated that nomograms were more practical and accurate for risk levels ranging from 2.5%–90% to 1%–78%. In summary, the nomogram model in this study is more accurate at predicting transfusion risk in patients undergoing spinal fusion surgery. In addition, its implementation can eliminate the need for unnecessary preoperative testing for T&S greatly improving clinical utility and providing significant cost savings with minimal disruption to clinical care, with a training set sensitivity of 78.2%, specificity of 86.7%, positive predictive value of 29.4% and negative predictive value of 98.2%, eliminating 2911 T&S tests (82.4%), resulting in a cost saving of RMB 349,320, but requiring 439 additional T&S tests. The validation set has a sensitivity of 75.2%, a specificity of 88.8%, a positive predictive value of 34.3%, and a negative predictive value of 97.9%, meaning that if the model were applied to the test set in clinical practice, it would eliminate 1276 T&S tests (84.2%), resulting in a cost saving of RMB 153,120, but requiring an additional 157 T&S tests. Ultimately, the need for T&S testing must be determined based on clinical judgment according to the patient's specific situation.

### Strengths and Limitations

To the best of our knowledge, this is the first study in Southwest China to develop a new predictive nomogram derived from a single high‐volume center to guide preoperative T&S testing in patients undergoing spinal fusion surgery. The constructed nomogram graphically displays all the critical factors and can be used to individually assess the risk of blood transfusion for spinal fusion. The model can aid clinical decision‐making, identify patients at high risk of transfusion, inform transfusion, reduce hospital costs, and reduce the environmental impact of blood collection.^34^

Nevertheless, this study has some limitations. Firstly, due to the study's retrospective design, the argument for causality is limited and there are also sampling and associated errors; however, the content selected and analyzed in this study are all traceable data from the hospital medical record system, thereby minimizing such errors. Secondly, the data of all patients were collected from the same hospital. Despite the good accuracy achieved by the nomogram model, there is still room for further prospective multicenter validation to verify and increase the reliability of the nomogram and further improve its clinical application. Third, the nomogram prediction model was only validated internally. Therefore, external validation is needed to enhance the predictive power of the nomogram model in the future. Finally, although the model validation results of this study are good, there are many factors that affect blood transfusion rates and these factors were not fully included in the analysis, which limits the usability of the model.

## Conclusions

In conclusion, this study showed that gender, age, outpatient admission, BMI, critical patient, operative time, heart failure, ESRD or CKD, anemia and coagulation defects were independent factors for postoperative or intraoperative blood transfusion in spinal fusion surgery. Furthermore, we used these factors to develop and validate a nomogram for transfusion risk in patients undergoing spinal fusion surgery according to the above factors. Validation showed that the model has a high precision and high discrimination in predicting transfusion and determining whether an individual requires transfusion based on our nomogram to selectively schedule preoperative T&S testing in appropriate patients, thus assisting clinicians in clinical decision‐making. However, external validation remains a future requirement.

## Conflict of Interest Statement

We declare that our research has been carried out without any commercial or financial relationships that might constitute a potential conflict of interest.

## Author Contribution

All authors had access to the study data and are responsible for the integrity and authenticity of the data. Study conception and design: Linghong Wu, Xiangtao Xie. Data collection: Xiaozhong Peng, Xianglong Zhuo, Guangwei Zhu. Data analysis and/or interpretation: Linghong Wu, Xiaozhong Peng, Xianglong Zhuo, Xiangtao Xie. Drafting of the original version: Linghong Wu, Xiaozhong Peng. Critical revision of important intellectual content:Xianglong Zhuo, Guangwei Zhu, Xiangtao Xie.

## Disclosures Statement

All authors declared no financial support or conflict‐of‐interest relationships. All of the authors meet the journal criteria for authorship, and have agreed to the publication of this manuscript.
